# Health Impacts of Increased Physical Activity from Changes in Transportation Infrastructure: Quantitative Estimates for Three Communities

**DOI:** 10.1155/2015/812325

**Published:** 2015-10-04

**Authors:** Theodore J. Mansfield, Jacqueline MacDonald Gibson

**Affiliations:** Gillings School of Global Public Health, University of North Carolina at Chapel Hill, 148A Rosenau Hall, CB No. 7431, Chapel Hill, NC 27599, USA

## Abstract

Recently, two quantitative tools have emerged for predicting the health impacts of projects that change population physical activity: the Health Economic Assessment Tool (HEAT) and Dynamic Modeling for Health Impact Assessment (DYNAMO-HIA). HEAT has been used to support health impact assessments of transportation infrastructure projects, but DYNAMO-HIA has not been previously employed for this purpose nor have the two tools been compared. To demonstrate the use of DYNAMO-HIA for supporting health impact assessments of transportation infrastructure projects, we employed the model in three communities (urban, suburban, and rural) in North Carolina. We also compared DYNAMO-HIA and HEAT predictions in the urban community. Using DYNAMO-HIA, we estimated benefit-cost ratios of 20.2 (95% C.I.: 8.7–30.6), 0.6 (0.3–0.9), and 4.7 (2.1–7.1) for the urban, suburban, and rural projects, respectively. For a 40-year time period, the HEAT predictions of deaths avoided by the urban infrastructure project were three times as high as DYNAMO-HIA's predictions due to HEAT's inability to account for changing population health characteristics over time. Quantitative health impact assessment coupled with economic valuation is a powerful tool for integrating health considerations into transportation decision-making. However, to avoid overestimating benefits, such quantitative HIAs should use dynamic, rather than static, approaches.

## 1. Introduction

In the United States, approximately 234,000 premature deaths are associated with physical inactivity each year [1]. The built environment influences walking and biking for transportation and, in turn, total physical activity [2, 3]. Many communities in the United States are designed in ways that do not support walking and biking, thereby contributing to low levels of physical activity [4]. Recently, transportation agencies across the United States have sought to integrate health considerations into decision-making [5, 6]. Health impact assessment (HIA) has emerged as a systematic framework for considering how decisions, such as modifications to the built environment, may impact public health and has informed a variety of decisions in the transportation sector [7, 8]. However, most transportation HIAs conducted to date have provided qualitative rather than quantitative estimates of health benefits arising from changes in physical activity (e.g., indicating that physical activity is expected to increase, without estimating the magnitude of the increase) [9]. Existing research links the built environment to physical activity levels and health outcomes, but quantitative models to predict the health impacts of modifications to the built environment remain poorly developed [10–12].

Within the past four years, two new tools to support quantitative HIAs have emerged. The first tool, the Health Economic Assessment Tool (HEAT) for cycling and walking, was introduced by the World Health Organization in 2011 [13]. More recently, the European Union Health Programme released the Dynamic Model for Health Impact Assessment (DYNAMO-HIA) [14]. These two tools employ fundamentally different methods; while DYNAMO-HIA is dynamic, capable of tracking changes in population health over many years, HEAT is static, providing health impact estimates for a single year. The HEAT method has been used in several HIAs of policies or projects to promote active transportation (walking or cycling instead of driving) [14]. DYNAMO-HIA has been applied to estimate the health impacts of a ban on alcohol imports in Sweden, smoking cessation in Great Britain, reduced salt intake in Europe, decreased smoking prevalence in Copenhagen, and body mass index reduction in Netherlands [15–18]. However, to our knowledge, DYNAMO-HIA has not yet been applied to predict the health impacts of increased physical activity arising from changes in the built environment. Further, the estimates from these two methods have not been compared.

To demonstrate the use of quantitative tools for estimating the health effects of physical activity in HIAs of the built environment, this paper describes quantitative HIAs of proposed changes to the built environment in three North Carolina communities. All three HIAs used DYNAMO-HIA to estimate the health effects of increased transportation walking time expected to arise due to modifications to the built environment. Changes in premature mortality, coronary heart disease (CHD), type 2 diabetes, hypertension, and stroke were estimated for each community. In addition, each HIA estimated the ratio of health benefits to expected project costs. For one of the case studies, we additionally compared results obtained from DYNAMO-HIA with those obtained from the HEAT model. Our objective in making this comparison was to determine whether the health impact estimates differ when using a dynamic approach (as in DYNAMO-HIA) as compared to a static approach (as in HEAT). We hypothesized that the static approach may overestimate health benefits by failing to account for overall improvements in population health from one year to the next and, as a result, estimating benefits in each year relative to a population for which no benefits have yet accrued. Our overall purpose was twofold: first, to demonstrate that quantitative tools in general may provide objective, evidence-based decision support within the HIA framework and, second, to provide insight into the advantages and disadvantages of emerging quantitative tools and methods to conduct HIAs.

The HIAs presented in this study were conducted as examples to support* WalkBikeNC*, a statewide bicycle and pedestrian plan developed by the North Carolina Department of Transportation (NCDOT) in 2013 [19].* WalkBikeNC* presents a unified policy framework to support active travel statewide, but it does not propose projects. Instead, specific bicycle and pedestrian infrastructure projects are planned and implemented by local authorities in accordance with* WalkBikeNC*. Such projects may be included in a range of local plans, including small-area plans, comprehensive transportation plans, and bicycle and pedestrian master plans. The three HIAs described in this paper consider pedestrian infrastructure improvements aligned with the policy framework established in* WalkBikeNC* at three planning scales: a small-area plan, a comprehensive plan, and a streetscape plan.

## 2. Materials and Methods

All three case studies followed the six steps of HIA proposed by the US National Research Council: (1) screening; (2) scoping; (3) assessment; (4) recommendations; (5) reporting; and (6) monitoring and evaluation [7]. The first two steps of HIA, screening and scoping, focus on identifying and characterizing health concerns and disparities in the community. The third step, assessment, explores how the decision to be made influences these concerns and disparities through qualitative understanding and/or quantitative modeling of causal pathways as understood in the scientific literature. The conclusions from the assessment stage inform the fourth stage, recommendations. Finally, reporting and monitoring and evaluation aim to engage stakeholders, hold decision-makers accountable, and evaluate the effectiveness of the decision in addressing identified health concerns at some point in the future. Because this paper focuses on improving the assessment stage through the application of quantitative methods, details of steps 4–6 are not presented; these details can be found elsewhere [19, 20]. Details on the screening and scoping stages are provided below, because these steps influenced the scope of the assessment phase.

### 2.1. Site Selection (Screening)

Case study sites were selected in coordination with NCDOT. In all three communities, the proposed changes to the built environment were included in adopted local plans but had not received funding as of October 2012 (when this project began). Projects were selected to provide variation across three dimensions: (1) development context (rural, suburban, and urban); (2) planning scale (corridor plan, small-area plan, and comprehensive plan); and (3) geographic region within North Carolina (Piedmont region, coastal region, mountain region). Table S1 and Figures S1–S3 in Supplementary Material available online at http://dx.doi.org/10.1155/2015/812325 provide maps, demographic data, and information about the changes to the built environment proposed for each project.

The first HIA is conducted on changes to the built environment proposed in the City of Raleigh's Blue Ridge Road Corridor (BRRC) small-area plan (*urban, small-area plan, Piedmont region*). The BRRC is located eight kilometers east of downtown Raleigh, the second-largest city in North Carolina and the state capital. The BRRC small-area plan is the result of a planning and visioning process to guide development in the corridor as it urbanizes. The plan includes dense, mixed-use land development, construction of a compact street network, and construction of additional pedestrian and bicycling facilities. We considered the effects on time spent walking for transportation and the resulting health outcomes if the plan were implemented in its entirety [21].

The second HIA is conducted on construction of new sidewalks in the town of Winterville as proposed in the Greenville Metropolitan Planning Organization's Bicycle and Pedestrian Master Plan (*suburban, comprehensive plan, *coastal* region*). This plan proposes both pedestrian and bicycle projects throughout the Greenville metropolitan area, a mid-size community in eastern North Carolina. We estimated the health impacts of building all sidewalks proposed in the plan within the municipal boundaries of Winterville, a suburban community on the outskirts of the Greenville region [22].

The third HIA is conducted on streetscape improvements proposed in the Town of Sparta's Downtown Streetscape Master Plan (*rural, corridor plan, mountain region*). Sparta is a prototypical rural main-street community, with a small, walkable downtown containing shops and services surrounded by low-density development. We estimated the health impacts of proposed improvements to the downtown streetscape, including improved sidewalks and street crossings [23].

### 2.2. Selection of Health Outcomes (Scoping)

Facilitated discussions with local decision-makers and residents in each community confirmed that existing transportation infrastructure (e.g., lack of sidewalks) and overall community design (e.g., lack of destinations within easy walking distance) limit opportunities for walking as a means of transportation. The potential health outcomes that could be affected if new, pedestrian-friendly infrastructure were in place and if, as a result, residents spent more time walking for transportation were then selected from a literature review. The literature review identified several health outcomes for which nonvigorous transportation physical activity has been shown to have a preventive effect: coronary heart disease (CHD), type 2 diabetes mellitus, hypertension, stroke, and premature mortality from all causes [24–27]. Additionally, these four diseases were identified as existing health concerns related to physical activity levels in each community.

### 2.3. Health Impacts Model (Assessment)

We used DYNAMO-HIA to estimate the health impacts of increased transportation physical activity in all three communities. We then additionally used a modified version of the HEAT model, implemented in* Analytica 4.5* (Lumina Decision Systems, Los Gatos, CA) in the BRRC. These two models and their data requirements are described in turn below.

**Figure 1:**
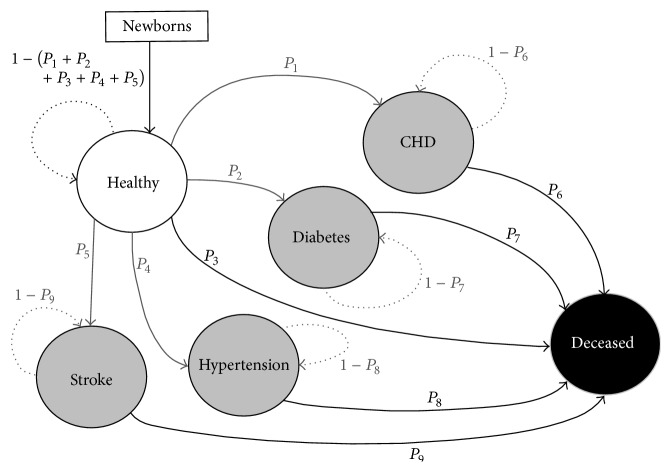
Schematic of DYNAMO-HIA model representing simulation of one time step for one scenario (reference or intervention). Each circle represents a population state. Solid lines represent possible transitions between states at each time step, whereas dotted lines represent staying in the same state during a time step. The variables *P*_1_–*P*_9_ represent transition probabilities between states.

DYNAMO-HIA is a dynamic health impacts model that employs Markov Chain modeling to estimate the effects of a health intervention on a population over time [15]. Conceptually, Markov Chain models divide a system into distinct groups of risk factor states linked by transition probabilities, which define the likelihood that a member of one group will transition to another group over time (Figure 1). The model moves forward in discrete one-year time steps, estimating the population in each group at time step using the previous group populations and transition probabilities between groups. To estimate the health impacts of an intervention that changes health behaviors, an intervention scenario is specified in which the probabilities of transitioning from a healthy to a diseased state (represented in Figure 1 as *P*_1_, *P*_2_, *P*_4_, and *P*_5_) or from a healthy or diseased state to death (*P*_3_ and *P*_6_–*P*_9_) are altered based on changes in the distribution of risk factors in the population (e.g., amount of time walking for transportation). As the model steps forward through time, changes in these transition probabilities affect the rate at which healthy individuals transition to diseased states and/or death. Alongside the intervention scenario, a baseline scenario is also specified in which transition probabilities are not affected by the intervention. Health impacts are estimated by comparing health outcomes between the two scenarios over time. DYNAMO-HIA requires a large amount of baseline health data: age- and sex-specific population distributions, mortality rates, disease prevalence, disease incidence rates, and risk factor prevalence. In the intervention scenario, a change in risk factor prevalence and/or a transition between risk factor states over time must also be specified. Finally, dose-response functions must be characterized for each health outcome of interest. DYNAMO-HIA is available free of charge (http://www.dynamo-hia.eu/) and may be installed on any Windows-based machine.

We developed DYNAMO-HIA models for each community. Each model included community-specific population and health data as described in Section 2.3.1. A baseline, “no-build” scenario and an intervention scenario were specified for each community. In the baseline scenarios, weekly time spent walking for transportation was taken from recent surveys as described in Section 2.3.3. In the intervention scenarios, studies linking proposed built environment changes in each community to increases in walking for transportation were used to estimate post-construction walking as described in Section 2.3.4. Relative risks linking time spent walking for transportation to modeled health outcomes were taken from epidemiological studies (Table 1). Health impacts were estimated by taking the difference in projected health outcomes between the two scenarios over time each year for 40 years.

To develop 95% confidence intervals for our health impact estimates, each model was run five times, changing relative risk parameters in the model to the upper and lower bound of the 95% confidence intervals reported in epidemiological studies in each iteration. The first model used central values for all relative risk parameters, the second model used the lower bound of the confidence interval for mortality and central values for all diseases, the third model used the upper bound of the confidence interval for mortality and central values for all diseases, the fourth model used lower bounds for all diseases and the central value for mortality, and the fifth model used upper bounds for all diseases and the central value for mortality. Varying each relative risk parameter in turn and rerunning each model enabled the construction of 95% confidence intervals for all of our results reflecting uncertainty in the relative risk parameters used; however, uncertainty in other model parameters (e.g., magnitude of changes in walking for transportation) is not reflected in these estimates. All confidence intervals reported throughout this paper were developed using this approach.

Unlike DYNAMO-HIA, the HEAT model is static: it estimates a fraction of cases of premature mortality that could be avoided if a population spent more time walking or cycling and assumes that this fraction is constant from year to year. That is, health benefits of increased activity do not accrue from year to year for a given individual. The WHO has made an online tool for automating these calculations (http://www.heatwalkingcycling.org/) available. In order to compare the results obtained with DYNAMO-HIA with those obtained using the HEAT model approach, we reconstructed the HEAT tool using* Analytica*. This reconstruction additionally includes morbidity, which is not included in the base HEAT model. Details of this reconstruction are provided elsewhere [28].

Like DYNAMO-HIA, our reconstructed version of the HEAT model requires baseline data on population size by age and sex, baseline death rates, baseline disease prevalence and incidence rates for each health outcome of interest, and relative risks linking each health outcome to a risk factor (in this case, walking for transportation). In addition, information about the time spent walking for transportation under current conditions and under the intervention scenario is needed. Sources for these data, used in both the DYNAMO-HIA models the reconstructed HEAT model in the BRRC, are described below.

#### 2.3.1. Baseline Population and Health Data

We estimated age- and sex-specific population distributions by applying county-level age and sex distributions to refine Census block-group data for each case study location (Figure S2) [29, 30]. Baseline death and birth rates were taken from county-level data obtained from the NC State Center for Health Statistics [31]. We developed age-specific prevalence functions for CHD, type 2 diabetes mellitus, hypertension, and stroke for each case study location by fitting second-order prevalence functions to data from the Behavioral Risk Factor Surveillance System (BRFSS) survey [32]. Disease prevalence data were not available stratified by both age and sex; thus, we stratified by age only and assumed identical prevalence functions for males and females. Incidence data are not available from the State Center for Health Statistics for the diseases considered in this study. Thus, incidence functions for each case study location were estimated using a differential equation-based method described in Brinks (see Supplementary Material, Section 2.1 and Table S4) [33].

#### 2.3.2. Relative Risks

Relative risks of each health outcome as a function of transportation walking were drawn from previous studies (summarized in Table 1). Categorical dose-response functions for type 2 diabetes mellitus and hypertension were taken from a study of US adults that used data from the National Health and Nutrition Examination Survey [26]. To our knowledge, no studies exist linking transportation physical activity levels to CHD or stroke risk in US adults; thus, relative risks were taken from two studies of a large cohort of Finnish adults [24, 25]. To estimate the relative risk of premature mortality as a function of time spent walking for transportation, a dose-response function derived in a recent meta-analysis was employed; this same function is used to calculate the relative risk of all-cause mortality in the HEAT model [13, 27]:

(1) $$\begin{array}{c} {RR}_{mortality}=0.89^{\left(y/168\right)}, \end{array}$$

where *y* is weekly minutes spent walking for transportation. We used (1) to estimate the relative risk of all-cause mortality for the same exposure categories used in studies linking walking for transportation to disease risk. Specifically, these studies grouped populations into three levels of time spent walking for transportation: a reference category (none), a low category (1–149 min/week), and a high category (150+ min/week). The high category reflects the Centers for Disease Control and Prevention (CDC) minimum recommendation for total adult physical activity [34]. Using (1), we calculated relative risks for all-cause mortality at the midpoint of the low transportation walking category (75 min/week) and at the low point of the high transportation walking category (150 min/week).

#### 2.3.3. Baseline Active Transportation Behavior

In Winterville and Sparta, we estimated baseline transportation physical activity using data from the 2009 North Carolina BRFSS survey [32]. In the BRRC, we used an active transportation survey conducted within the neighborhood in 2012 utilizing a widely used and validated physical activity questionnaire [20, 28, 35]. Responses to these surveys were recategorized according to the CDC physical activity categories described above.

#### 2.3.4. Estimating Changes in Active Transportation Behavior

Due to differences in data availability and the nature of the plans considered, different methods were used in each case study community to estimate how changes in the built environment are expected to affect transportation physical activity.

The method for estimating changes in walking time if the BRRC small-area plan were implemented is described in detail elsewhere [20, 28]. Briefly, because multiple built environment changes are proposed in addition to pedestrian infrastructure improvements, the net effect of all of these changes on transportation walking is estimated using a multidimensional walkability index that links intersection density, population density, land-use diversity, and retail floor area ratio to walking for transportation [36]. The walkability index is calculated from

(2) Walkability  Score=2×Zintersetion+Zresidential+ZFAR+Zland-use,

where *Z* variables represent normalized versions of intersection density (*Z*_intersetion_), the number of intersections divided by land area; residential density (*Z*_residential_), the number of housing units divided by the residential land area; retail floor area (*Z*_FAR_), the square footage of retail floor area divided by the square footage of land devoted to retail use; and land-use diversity (*Z*_land-use_), computed as described in Cervero and Kockelman [37]. Previous studies that have linked transportation walking time to the walkability score were then used to estimate the increase in time spent walking as a result of the increase in walkability score that would occur if the small-area plan were fully implemented [28, 38].

In Winterville, the proposed changes to the built environment consist solely of new sidewalk construction. Thus, a relationship linking sidewalk density to transportation walking was used to estimate changes in transportation physical activity. A 1 km/km^2^ increase in sidewalk density is associated with an increase in the odds of an individual having taken a walking trip in the previous week by 2.3 percent [39]. Thus, the odds ratio of walking before and after construction may be expressed as:

(3) $$\begin{array}{c} \frac{O_{W,after}}{O_{W,before}}=1.023^{\left(D_{s,after}-D_{s,before}\right)}. \end{array}$$

*O*_*W*,before_ is the odds of walking given the density of sidewalks before construction, *D*_*s*,before_ (km/km^2^), and *O*_*W*,after_ is the odds of walking given the density of sidewalks after construction, *D*_*s*,after_ (km/km^2^). Rearranging (3) and expressing in terms of probabilities, this becomes:

(4) $$\begin{array}{c} \frac{P_{W,after}}{\left(1-P_{W,after}\right)}=\frac{{P_{W,before}1.023}^{\left(D_{s,after}-D_{s,before}\right)}}{\left(1-P_{W,before}\right)}. \end{array}$$

*P*_*W*,*after*_ is the probability that an individual takes at least one walk trip per week after construction, and *P*_*W*,before_ is the probability that an individual has taken a walking trip in the past week before construction, assumed to be equal to the proportion of the population reporting any walking in the BRFSS. We iteratively solved for *P*_*W*,after_ and adjusted the proportion of non-walkers in the population accordingly. We assumed that new walkers were distributed between the low- and high-walk-time categories in the same manner as walkers were distributed between these two categories before construction.

In Sparta, we used changes in a composite pedestrian environment factor (PEF)—which includes sidewalk quality, ease of street crossings, topography, and density of the street grid—to estimate changes in average weekly walking distance [40]. Each subcategory is assessed on a 3-point scale; the PEF is calculated by adding these four subcategory scores and transforming the result into an ordinal variable (low, medium, or high). After construction of streetscape improvement in Sparta, sidewalk quality and ease of street crossings would improve significantly while topography and the configuration of the street network would remain unchanged. Therefore, we assumed that the sidewalk quality and ease of street crossings subcategories would change from 1 (current conditions) to 3 (post-construction), while the topography and street grid density would remain unchanged. This change in subscores would change the PEF from low to medium. In turn, per-capita weekly walking distance would increase by 0.92 kilometers [40]. Assuming a typical walking speed of 4 kilometers per hour, per-capita transportation walking time would increase by 13.6 minutes per week, on average [41]. Because this relationship was derived in an urban setting using small geographies, while Sparta is a rural town, we assumed that only individuals living within a 0.4-kilometer buffer of the proposed improvements (25% of the population) would increase their walking. We increased the percentage of population in each walking time bin proportionally so that the average per-capita walking time for individuals living within 0.4 kilometers of the proposed improvements equaled to the preconstruction average plus 13.6 minutes.

#### 2.3.5. Economic Valuation

To compare the benefits of estimated health impacts to project costs, we applied economic valuations to each health outcome considered. For mortality, we used the value of a statistical life suggested by the United States Department of Transportation (USDOT) in 2013, $9.1 M USD per avoided premature death [42]. For each disease, we used yearly disease costs estimated by the Milken Institute that combine treatment costs and indirect costs from productivity losses resulting from lost workdays and reduced presenteeism (in Supplementary Material, Table S7) [43]. For the BRRC and Winterville, we estimated project costs using average bid data for North Carolina ($89.57 per linear meter of sidewalk; $142.08 and $150.70 per square meter of poured concrete sidewalk and curb and gutter, resp.) [44]. For Sparta, we used the cost estimate provided in the plan, $686,157 USD [23]. Ongoing maintenance costs are not considered. Benefits and costs were discounted to the present using a 5% discount rate per USDOT guidance [45]. A sensitivity analysis was conducted using 3.5% and 7% discount rates based on guidance from the United States Office of Management and Budget and NCDOT, respectively (in Supplementary Material, Figure S5) [45, 46].

## 3. Results

### 3.1. Health Outcomes

**Figure 2:**
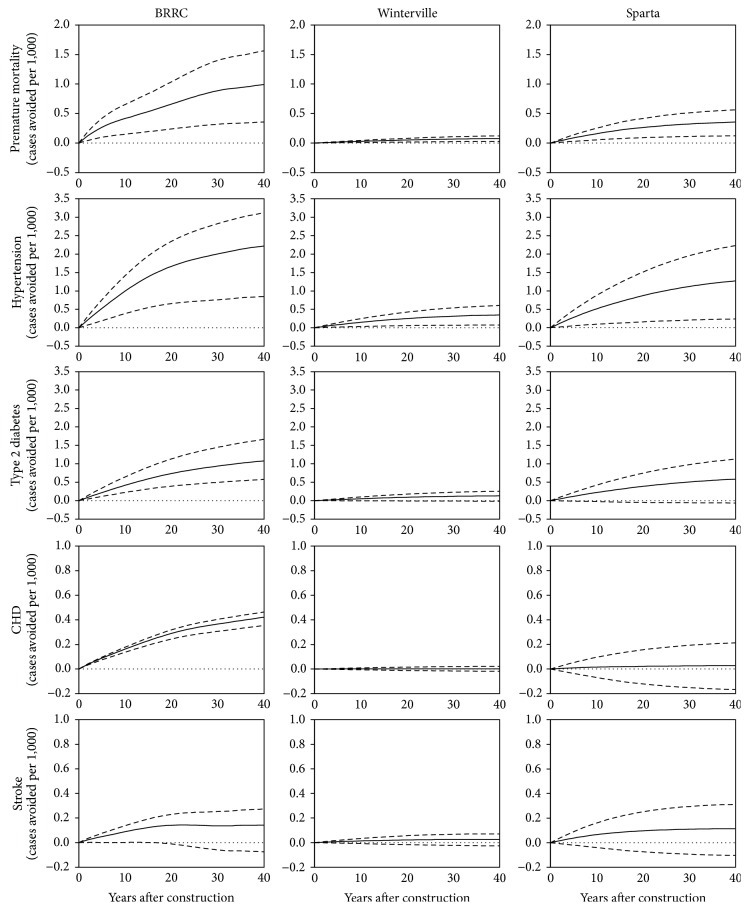
Estimated health impacts per 1,000 persons for each community (solid lines), with 95% confidence intervals reflecting uncertainty in relative risk parameters (dashed lines).

To estimate the health impacts of built environment changes in each community, we used DYNAMO-HIA to predict changes in premature mortality and incidence of CHD, type 2 diabetes, hypertension, and stroke over 40 years due to increased walking for transportation. In the BRRC, DYNAMO-HIA estimates a significant reduction in premature all-cause mortality as well as significant preventive effects for hypertension, type 2 diabetes mellitus, and CHD (Figure 2). In Sparta, significant reductions in premature mortality, cases of hypertension, and cases of type 2 diabetes mellitus are estimated; however, estimated effects on avoided cases of CHD are minimal. In Winterville, DYNAMO-HIA estimates small, yet significant, reductions in premature mortality and cases of hypertension and minimal effects on type 2 diabetes and CHD. Across all sites, no significant reductions in cases of stroke are estimated. The total population benefits of avoided mortality and the prevention of hypertension and type 2 diabetes accrue over time but demonstrate diminishing returns (Figure 2, Table 2). For example, DYNAMO-HIA estimates that the cumulative number of premature deaths avoided in the BRRC will increase from 4.9 (1.8–7.7) ten years after construction to 14 (5.2–23) 40 years after construction (Table 2). Similarly, within ten years of construction, an estimated 12 (4.5–17) and 4.9 (2.6–7.6) cases of hypertension and type 2 diabetes will have been prevented, and these numbers are expected to increase to 32 (12–45) and 16 (8.3–24) within 40 years. Generally, health outcomes for which a strong preventive effect is demonstrated in the literature and for which baseline community prevalence is high (e.g., hypertension) are most influenced by increases in transportation physical activity.

Comparing across sites, DYNAMO-HIA estimates stronger preventive effects on a per-capita basis in the BRRC and Sparta than in Winterville (Figure 2). For example, the cumulative cases of premature mortality prevented by year 40 are 0.99 and 0.36 per 1,000 people in the BRRC and Sparta, respectively, as compared to 0.08 per 1,000 people in Winterville. This result occurs because the proposed changes to the built environment in the BRRC and Sparta are estimated to increase transportation walking more in the BRRC and in Sparta than in Winterville (Table 2). For example, the average time spent walking per week is expected to increase by 17 minutes in the BRRC and 2.2 minutes in Sparta, in comparison to a smaller increase of 0.7 minutes per week in Winterville (Table 2). Additionally, a preventive effect on CHD is only estimated in the BRRC. As shown in Table 1, the preventive effect of walking for transportation on CHD is strong only for females in the highest physical activity category. The population in the BRRC has a greater proportion of women compared to the other two sites (in Supplementary Material, Figure S4) and a greater predicted change in the proportion of the population walking more than 150 minutes per week for transportation (Table 2); thus, the effect of increased transportation walking on avoided cases of CHD is significant in the BRRC but not in the other two sites.

### 3.2. Economic Valuation

To estimate the economic value of health benefits in each community, we multiplied projected avoided deaths and avoided disease cases per year by their respective economic values. The economic value of estimated health benefits exceeds project construction costs within one year in the BRRC and within three years in Sparta (Table 2) assuming a 5% discount rate. Over the 40-year time period considered, the benefit-cost ratios in the BRRC and Sparta are 20.2 (8.7–30.6) and 4.7 (2.1–7.1), respectively. However, the present value of the health benefits in Winterville is less than the estimated project costs: the benefit-to-cost ratio in Winterville over 40 years is 0.6 (0.3–0.9) (Table 2). This latter finding results from the design of the Winterville project and the population density in that community; while significant sidewalk construction is proposed, the new sidewalks will be spread over a very large area of relatively low population density, dampening the potential behavioral impact. The net present value of the BRRC and Sparta projects remains positive even when considering a higher discount rate (7%) and remains negative in Winterville even when considering a lower discount rate (3.5%) (in Supplementary Material, Figure S5).

In all communities, health benefits are overwhelmingly driven by avoided premature mortality (Figure S5). Avoided premature mortality constitutes 92%, 86%, and 89% of the total net present value of health benefits over 40 years in the BRRC, Winterville, and Sparta, respectively. This result occurs due to the much higher value placed on an avoided premature death, in comparison to the value placed on avoided chronic disease cases (in Supplementary Material, Table S7).

### 3.3. Comparison of DYNAMO-HIA and HEAT

**Figure 3:**
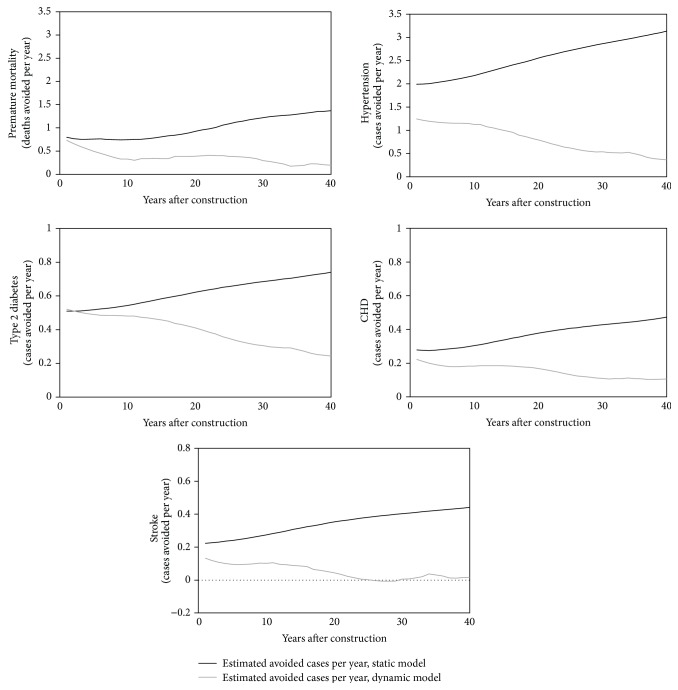
Estimated health impacts per year obtained using the HEAT (static) model (solid black lines) and DYNAMO-HIA (dynamic) model (solid grey lines) for the BRRC case study.

**Figure 4:**
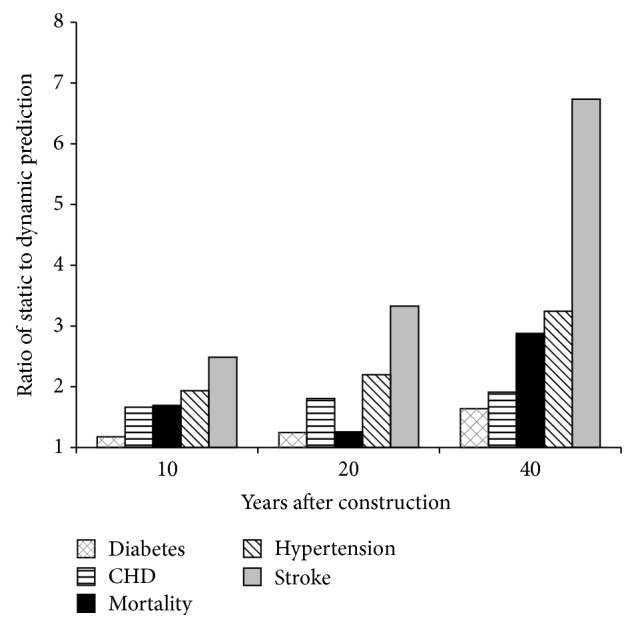
Ratio of cumulative health impact estimates from HEAT (static) and DYNAMO-HIA (dynamic) models at 10, 20, and 40 years after construction.

To compare the dynamic approach used in DYNAMO-HIA and the static approach used in the HEAT model, we re-estimated health impacts in the BRRC using our reconstructed HEAT model and compared these findings to impacts estimated by our DYNAMO-HIA model. For all health outcomes considered, the HEAT model estimates a higher number of avoided cases per year than the DYNAMO-HIA model (Figure 3). The difference between the two approaches increases with time (Figure 3). When considering the cumulative health impacts over multiple years, the differences in the two approaches become substantial (Figure 4). The reconstructed HEAT model estimates that 41 premature deaths would be prevented over 40 years—2.9 times as many deaths averted as predicted by the DYNAMO-HIA model. Similarly, central estimates of avoided hypertension, type 2 diabetes, CHD, and stroke increase by factors of 3.3, 1.6, 2.5, and 6.7 when using the static approach, in comparison to the dynamic approach (Figure 4).

The static approach overestimates health benefits by failing to account for changing disease prevalence over time. In the static model, avoided cases for each year are estimated for the population as a whole without accounting for population disease prevalence. In contrast, the dynamic model removes individuals who develop a disease from the population that is able to avoid a new case in subsequent years (i.e., individuals who develop a disease transition to diseased states (Figure 1), after which they are not included in estimations of new avoided cases). Additionally, the dynamic model references data from the previous year in estimating benefits for a given year whereas the static model has no memory of population health data in the previous year. Thus, relative to the dynamic model, the static model overestimates benefits in the future because it fails to account for changes in disease prevalence over time. In other words, the dynamic model is able to incrementally approach a new steady state in which an intervention has shifted disease incidence functions downwards for a portion of the population; once this steady state is reached, new benefits no longer accrue as lower risk individuals delay the onset of disease but do not completely avoid disease over time. Once these individuals transition into a diseased state, they are no longer included in avoided cases calculations. Static models, however, do not approach a new steady state because benefits are always calculated relative to a population in which no benefits have been accrued and disease prevalence is not accounted for. Thus, benefits will continue to accrue beyond the point at which the dynamic model reaches a new steady state. As a result, the static model increasingly overestimates benefits over time relative to the dynamic model. This behavior is illustrated in Figure 3; at each time step, the rate of change in avoided cases of type 2 diabetes stays relatively stable for the static model, increasing slightly as the population grows over time. In the dynamic model, the rate of change in the number of cases avoided decreases over time as the model approaches steady state in which all individuals who walk more have a decreased risk, but still some risk, for developing type 2 diabetes throughout their lifetimes (Figure 3).

## 4. Discussion

Using the dynamic DYNAMO-HIA tool, we predicted that the health benefits of changes to the built environment that support walking for transportation would exceed construction costs in two of the three case study communities. In the urban BRRC neighborhood, the benefit-cost ratio of changes to the built environment that would increase walkability was estimated to be 20 over 40 years. In the small rural town of Sparta, the benefit-cost ratio of proposed improvements to the downtown streetscape reached 4.7 over 40 years. In contrast, the benefit-cost ratio of constructing proposed sidewalks in suburban Winterville reached only 0.6 over 40 years. In addition, our comparison of estimates from the reconstructed HEAT model and estimates from the DYNAMO-HIA model showed that the static approach tends to over-predict benefits when considering effects over multiple years. Thus, if sufficient data and capacity exist, dynamic tools such as DYNAMO-HIA should be used rather than static tools to estimate the health impacts of policies and projects that increase transportation physical activity.

### 4.1. Comparison with Recent Active Transportation HIAs

A number of transportation HIAs using a range of modeling techniques to link changes in the built environment to health benefits from increased transportation physical activity have been completed in recent years [14]. To our knowledge, only one example of a dynamic model used to estimate the health benefits of built environment changes exists: a system dynamics model was used in an HIA of large-scale bicycle infrastructure construction in Auckland, New Zealand [47]. This model linked bicycle infrastructure investment scenarios to changes in the perceived safety of bicycling to work and resulting mode shifts to bicycle commuting. Health impacts were then estimated for resulting changes in bicycle crash risk, air pollution exposure, and physical activity levels. Bicycle mode shares were predicted for several investment scenarios, including a business-as-usual scenario. A relative risk function comparing cyclists to non-cyclists was used to estimate changes in mortality from increased physical activity for each scenario over time. Benefit-cost ratios ranged from 6 to 24, driven largely by the value of prevented premature mortality resulting from increased physical activity [47].

A number of HIAs using static models, including HEAT, have also recently been performed. A study in Dane County, Wisconsin, estimated a benefit-cost ratio of 1.7 for a hypothetical countywide sidewalk construction project. The study used a regression model to link sidewalk presence to time spent walking and biking for transportation. The results of this model were used to estimate transportation physical activity given sidewalk construction across the county. Increased physical activity was then linked to reduced weight gain and ultimately reduced costs associated with obesity using a static model [48]. An HIA of the construction of a bicycle path in Dublin, Ireland, estimated benefit-cost ratios ranging from 2.2 to 11.8. This HIA used a survey to estimate increased bicycling to work after construction and the HEAT model to estimate health and economic benefits [49]. Finally, an assessment in Portland, Oregon, used a traffic demand model to estimate increased bicycle commuting due to past and planned investments in bicycle infrastructure throughout the city. Using the HEAT model to estimate benefits from resulting increases in physical activity, benefit-cost ratios ranged from 20 to 53 [50]. As in our study, avoided premature mortality dominated the monetary value of the health benefits of increased physical activity (Figure S5).

Previous studies have found benefit-cost ratios for changes in the built environment that support walking and biking for transportation ranging 1.7 to 53. Our results are within this range for the BRRC and Sparta but not in Winterville. The population density in Winterville may be too low for the proposed improvements to be economically viable when considering health benefits alone. This finding demonstrates that the health benefits of changes in the built environment that increase physical activity may not always exceed project costs. Thus, quantitative HIA may be an important tool for prioritizing investments to maximize the overall value of health benefits.

As HIA for active transportation projects and policies is refined, it will be important to consider differential treatment effects for different age groups and to include social equity considerations [14]. Physical activity may have a stronger preventive effect for older individuals, and many countries worldwide are seeing shifts in population distribution towards older age groups. The dynamic model used in this assessment is able to easily incorporate age-specific dose-response information, if available. The usefulness of such stratifications is demonstrated in our estimates for CHD: due to differences in population characteristics and predicted changes in behavior across sites, we estimate reduced incidence of CHD in the BRRC but not in Sparta or Winterville. This difference is driven by differential treatment effects at higher doses of transportation walking for men and women (Table 1). To increase the consideration of social equity in transportation HIA, scalable models are needed. Using the DYNAMO-HIA model at three different scales, we provide evidence that quantitative assessment methods are robust across scales. If modeling methods are robust at different scales, a series of neighborhood-scale models may be used to compare the health impacts of transportation decisions in neighborhoods with different socioeconomic conditions and may reveal disproportionate impacts. Such an application could better inform investments in active transportation infrastructure to address social equity concerns.

In sum, previous studies provide strong evidence that built environment changes meaningfully impact health outcomes and are often quite economically advantageous. Our application of a novel dynamic model yields findings consistent with the existing literature, building the robustness of the link between the built environment, physical activity, and health benefits. Further, we demonstrate that dynamic models may be applied across a variety of scales and are able to incorporate differential treatment effects for different age groups and for men and women. Thus, dynamic models may help address identified limitations of transportation HIA in practice.

### 4.2. Limitations

Our estimates of post-construction physical activity do not consider activity substitution (i.e., reducing other activities after increasing transportation physical activity) or self-selection (i.e., more active individuals may be more likely to increase transportation physical activity). However, longitudinal evidence suggests that activity substitution is minimal, and increases in physical activity remain when self-selection is accounted for [51–53]. In addition, our estimates exclude potential increases in physical activity from walking for leisure and from bicycling and, in this regard, could underestimate health benefits.

Additionally, we consider only one health pathway (physical activity), while transportation influences health in other ways, including exposure to air pollution and crash risk. Other health pathways may respond to built environment changes in opposite directions and with different magnitudes. For example, compact urban forms may increase physical activity but also increase exposure to air pollution [54]. A recent HIA in London found health benefits from increased physical activity but also negative health impacts from increased exposure to air pollution and elevated crash risk for active commuters [55]. However, recent HIAs of active transportation consistently find changes in physical activity to be the largest contributor to estimated health impacts [14].

While DYNAMO-HIA is able to use continuous relative risk functions, continuous prevalence data are also required when doing so and must be characterized using the mean, standard deviation, and skewness of the distribution. Baseline distributions of walking for transportation were noncontinuous (taken from categorical survey responses) and difficult to characterize as continuous distributions due to excess zeroes. Further, continuous dose-response functions were not available linking walking for transportation with CHD, type 2 diabetes, hypertension, or stroke. To overcome these difficulties, the model uses a discrete dose-response function that caps health benefits at 150 minutes of transportation physical activity per week. As a result, the model may underestimate benefits for those accruing more than 150 minutes of transportation physical activity per week. To analyze the potential magnitude of this underestimation, we recomputed the static (HEAT) model predicted mortality reduction using a continuous dose-response function combined with categorical prevalence data using smaller bins (i.e., divided into eleven categories of weekly time spent walking for transportation). The latter model estimates an additional 26 (+63%) avoided deaths after 40 years. However, since both these models are prone to overestimation, this difference may be artificially inflated.

This paper considered only three communities in North Carolina. While representing a range of urban development contexts (rural, suburban, and urban), all three communities had low baseline levels of transportation physical activity and limited public transit service. Further, community-specific disease prevalence and incidence may reflect population characteristics specific to North Carolina. Thus, our findings concerning the relative costs and benefits of the planned infrastructure investments in these three communities may not generalize to highly urban settings with higher baseline levels of transportation physical activity, higher levels of public transit usage, and/or different demographic characteristics than North Carolina. However, the differences revealed comparing estimates from DYNAMO-HIA and the HEAT model stem from the different structures of the modeling approaches themselves and thus may be generalizable across communities of many types.

Finally, disease prevalence and incidence are estimated using county data. However, these data are identical in the baseline and intervention scenarios so any resulting bias is likely minimal.

## 5. Conclusion

Using DYNAMO-HIA to conduct three quantitative HIAs, we demonstrated that investments in infrastructure that supports active transportation may have meaningful impacts on health outcomes via increased transportation physical activity. These health outcomes may also have considerable financial implications: in two of the three cases, the benefits of avoided disease and premature mortality alone exceeded construction costs.

Dynamic health impact models, such as DYNAMO-HIA, offer significant advantages over static models, such as HEAT. Static models may overestimate health benefits by failing to account for changing population health characteristics over time. However, it may be difficult to implement continuous relative risk functions using existing dynamic modeling tools if baseline exposure information is difficult to characterize as continuous distributions or if continuous dose-response information is available only for certain health outcomes. If continuous dose-response functions are discretized into just a few categories, the benefits of physical activity may be underestimated for individuals who are very physically active. Providing greater flexibility in characterizing exposure or allowing continuous dose-response functions to be used alongside categorical exposure data in existing tools would address this shortcoming in practice. Overall, the advantages of dynamic models outweigh the current limitations of available tools.

Quantitative HIA is a feasible tool for objective, evidence-based decision support linking health outcomes to increased—or decreased—physical activity resulting from changes in the built environment. Transportation decision-makers routinely use models to estimate congestion reduction and improvement in traffic safety and translate these outcomes into monetary benefits [56]. Thus, quantitative HIA combined with economic valuation enables the health benefits of increased transportation physical activity from changes in the built environment to be considered alongside traditional transportation metrics. As transportation agencies search for ways to better integrate health considerations into transportation decision-making, quantitative HIA fills a critical gap, translating investment in infrastructure that supports active travel into a metric that enables direct comparison with other types of projects. Further, quantitative assessments of competing built environment risks, such as physical activity, air pollution, and traffic fatalities, may help align larger planning efforts (e.g., comprehensive plans) with health goals by comparing the public health impacts of alternative future scenarios. Using three cases across North Carolina, we demonstrated that quantitative models linking built environment changes to physical activity and health impacts are feasible, provide meaningful results to decision-makers, and may help prioritize resources in pursuit of public health goals.

## Supplementary Material

The Supplementary Material includes four sections. Section 1 provides additional details on each community, descriptions of proposed pedestrian infrastructure improvement projects, and summaries of community meetings. Section 2 presents the procedure used to estimate disease incidence functions and provides additional detail on baseline health data. Section 3 summarizes the surveys used to characterize preconstruction walking for transportation. Finally, Section 4 provides the economic values of health outcomes used in analysis and plots of economic valuations over time for each community.

## Figures and Tables

**Table 1:** Relative risks.

Health outcome	Sex	Low category (1–149 minutes' walking for transportation per week)	High category (150+ minutes' walking for transportation per week)
All-cause mortality [23]	Combined	0.95 (0.98–0.92)^a^	0.90 (0.96–0.85)

CHD [24]	Male Female	0.99 (1.08–0.91)^c^ 0.95 (1.08–0.83)^c^	0.99 (1.10–0.90)^c^ 0.80 (0.92–0.69)^c^

Type 2 diabetes [26]	Combined	0.77 (1.02–0.58)^b^	0.69 (0.88–0.54)^b^

Hypertension [26]	Combined	0.76 (0.94–0.61)^b^	0.69 (0.83–0.58)^b^

Stroke [25]	Male Female	0.94 (1.06–0.83)^c^ 0.88 (1.01–0.77)^c^	0.88 (1.02–0.77)^c^ 0.87 (1.01–0.75)^c^

^a^95% confidence interval shown for all relative risks.

^b^Adjusted for race, education, income, and smoking status.

^c^Adjusted for education, smoking status, alcohol consumption, body mass index, systolic blood pressure, cholesterol, history of diabetes, and occupational and leisure-time physical activity.

**Table 2:** Summary of findings, with 95% confidence intervals based on uncertainty in relative risk parameters.

Built environment variables	BRRC			Winterville			Sparta		
	Before	After	Change	Before	After	Change	Before	After	Change
Walkability score	−3.61	0.96	**+4.57**	—	—	—	—	—	—
Sidewalk density (km/km^2^)	—	—	—	0.8	3.8	**+3.0**	—	—	—
PEF (categorical)	—	—	—	—	—	—	Low	Medium	**+1**

Walking outcomes^a^	Before	After	Change	Before	After	Change	Before	After	Change

No walking (percent)	40.7%	40.7%	**0%**	84.3%	83.4%	−**0.9%**	85.4%	82.4%	−**3.0%**
1–149 min/week (percent)	41.5%	21.2%	−**20.3%**	12.3%	12.9%	**+0.6%**	12.1%	14.6%	**+2.5%**
150+ min/week (percent)	17.8%	38.1%	**+20.3%**	3.4%	3.6%	**+0.2%**	2.5%	3.0%	**+0.5%**
Ave. walk time (min/week)	13.1	30.4	**+17**	12.5	13.2	**+0.7**	10.4	12.6	**+2.2**

Health outcomes^a^	Years after construction			Years after construction			Years after construction		
	10	20	40	10	20	40	10	20	40

Avoided premature mortality	4.9 (1.8–7.7)	8.5 (3.1–13.3)	14.3 (5.2–22.6)	0.3 (0.1–0.5)	0.5 (0.2–0.9)	0.9 (0.3–1.4)	0.3 (0.1–0.4)	0.4 (0.2–0.7)	0.5 (0.2–0.8)
Avoided cases of CHD	1.9 (1.6–2.1)	3.7 (3.1–4.1)	6.1 (5.1–6.7)	0.0 (−0.1–0.1)	0.0 (−0.1–0.2)	0.0 (−0.2–0.3)	0.0 (−0.1–0.2)	0.0 (−0.2–0.3)	0.0 (−0.2–0.3)
Avoided cases of type 2 diabetes	4.9 (2.6–7.6)	9.4 (5.1–14.5)	15.6 (8.3–24.1)	0.5 (0.0–1.0)	1.0 (−0.1–1.9)	1.5 (−0.2–2.9)	0.4 (0.0–0.7)	0.6 (−0.1–1.2)	0.8 (−0.1–1.6)
Avoided cases of hypertension	11.8 (4.5–16.7)	21.4 (8.4–30.1)	32.1 (12.3–45.1)	1.5 (0.4–2.5)	2.7 (0.6–4.5)	4.0 (0.9–6.9)	0.9 (0.2–1.5)	1.4 (0.3–2.4)	1.8 (0.4–3.2)
Avoided cases of stroke	1.1 (0.0–1.6)	1.8 (−0.1–2.9)	2.1 (−1.1–4.0)	0.1 (−0.1–0.3)	0.2 (−0.2–0.6)	0.3 (−0.3–0.8)	0.1 (−0.1–0.3)	0.2 (−0.1–0.4)	0.2 (−0.2–0.5)

Economic outcomes^b,c^	Years after construction			Years after construction			Years after construction		
	10	20	40	10	20	40	10	20	40

Net present value (2012 USD)	33.4M (10.8–53.7)	50.4M (18.4–79.0)	66.8M (26.8–103)	−5.1M (−6.5–−3.9)	−3.9M (−5.9–−2.1)	−2.9M (−5.3–−0.6)	1.4M (0.1–2.5)	2.2M (0.5–3.7)	2.6M (0.7–4.2)
Benefit-cost ratio	10.6 (4.1–16.5)	15.5 (6.3–23.7)	20.2 (8.7–30.6)	0.3 (0.1–0.5)	0.5 (0.2–0.7)	0.6 (0.3–0.9)	3.0 (1.1–4.6)	4.1 (1.7–6.3)	4.7 (2.1–7.1)

Time for B : C to exceed 1	1 year (1-2 years)			Benefits do not exceed costs			3 years (2–9 years)		

^a^Estimates of walking for transportation after construction in Winterville do not add to 100% due to rounding.

^b^For all health and economic outcomes, 95% confidence intervals are estimated using the lower and upper bounds of the relative risk parameters as noted in Table 1.

^c^5% discount rate assumed.
