# Supplementary material for: Health Impacts of Increased Physical Activity from Changes in Transportation Infrastructure: Quantitative Estimates for Three Communities
Source: Biomed Res Int. 2015 Oct 4;2015:812325. doi: 10.1155/2015/812325 (PMC4609517; doi:10.1155/2015/812325)

## Supplementary Material

### S.1 Additional Case Study Information

Descriptive information for each case study location is summarized in Table S1. Summary information for meetings held in each community are presented in Table S2 (scoping meetings) and Table S3 (post-analysis meetings). Age- and sex-specific population distributions for each community are provided in Figure S4.

### Blue Ridge Road Project, Raleigh, NC

A community visioning and planning effort developed a small area plan for the Blue Ridge Road neighborhood, located in a currently suburban portion of Raleigh, NC. The small area plan includes significant land-use changes, construction of new sidewalks, and streetscape improvements (Figure S2). We consider the impact of new sidewalks proposed in the plan compared to a no-build scenario.

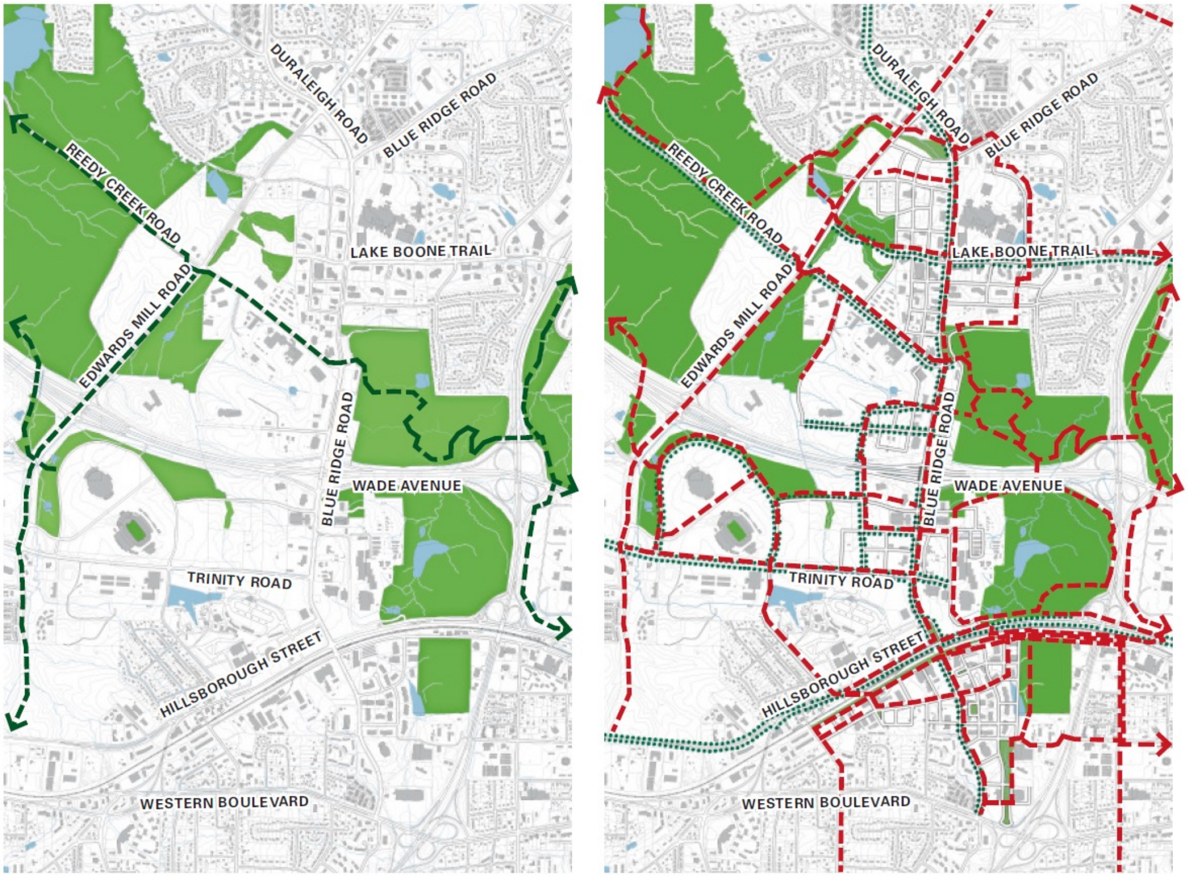


**Figure S2.** BRRC existing open space and trails (*left*) and proposed open space, trails, and improved sidewalks (*right*)

**Greenville MPO Bicycle and Pedestrian Master Plan, Winterville, NC**

In 2011, the Greenville MPO completed a Bicycle and Pedestrian Master Plan for the Greenville Metropolitan Area, which includes Winterville. We consider the impact of building out the pedestrian network as specified in the plan compared to a no-build scenario (Figure S1).

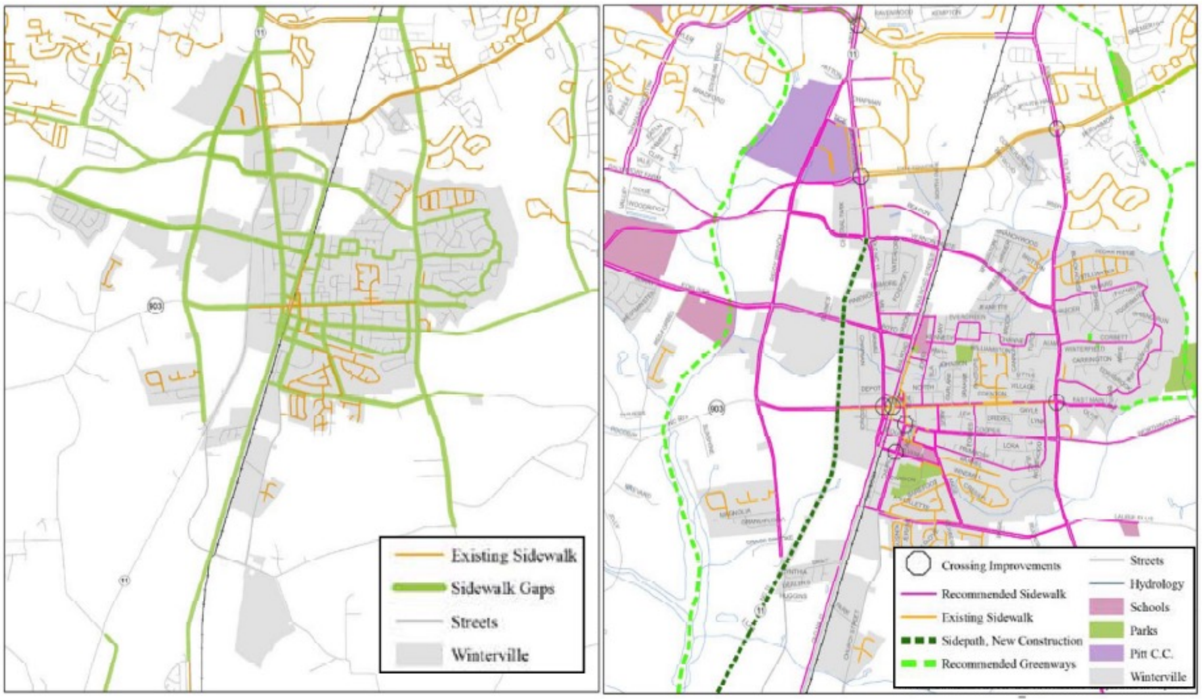


**Figure S1.** Winterville existing pedestrian facilities (*left*) and proposed improvements (*right*)

**Downtown Streetscape Master Plan, Sparta, NC**

In 2012, the town of Sparta, NC completed a Downtown Streetscape Strategy, which proposes a number of improvements to the pedestrian environment in downtown. We conducted an HIA on the implementation of the plan and compared the results to the status quo scenario. The project contains streetscape and street crossing improvements along Main Street, which runs through downtown Sparta, as well as complementary improvements to several side streets (Figure 3).

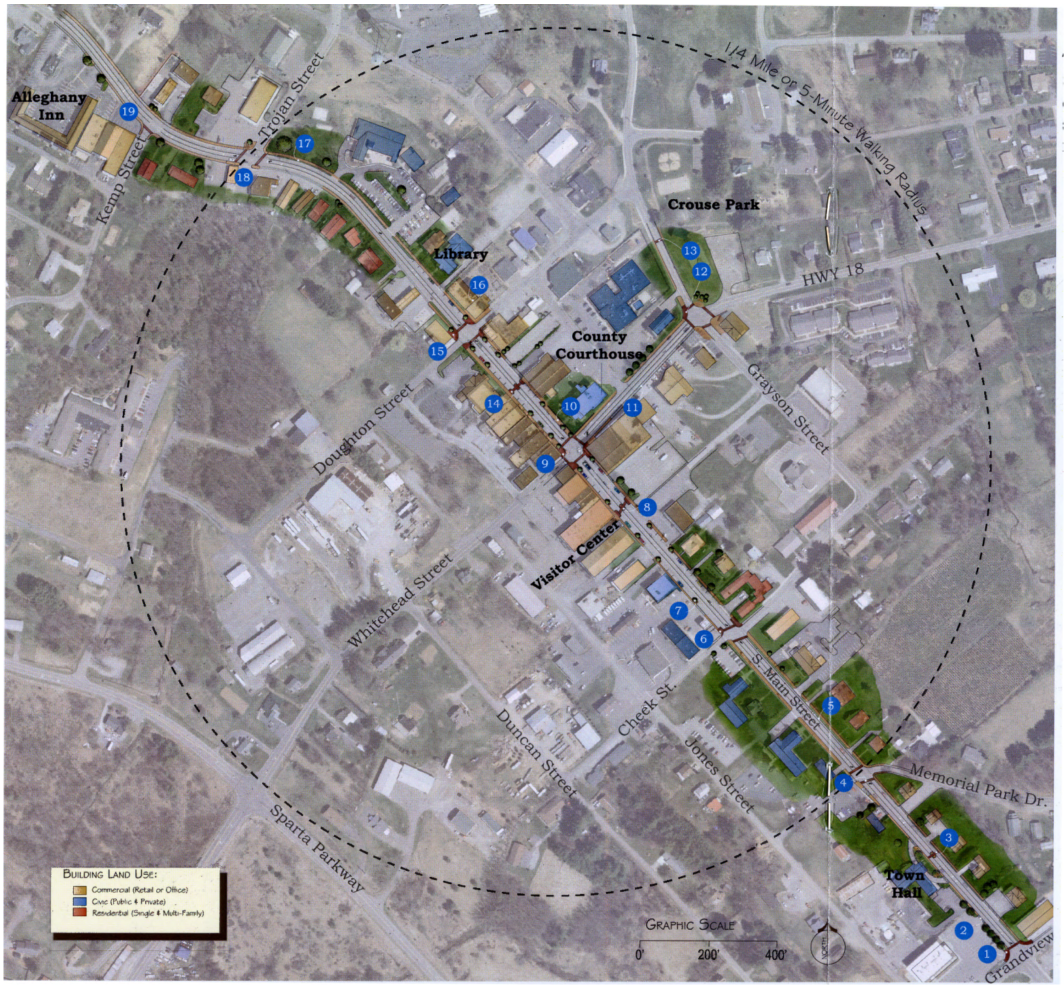


**Figure S3.** Sparta proposed downtown streetscape improvements

**Community Context**

Descriptive statistics for each case study location is summarized in Table S1. Summary information for meetings held in each community are presented in Table S2 (scoping meetings) and Table S3 (post-analysis meetings). Age- and sex-specific population distributions for each community are provided in Figure S4.

**Table S1.** Case Study Location Characteristics

| | BRRC | Winterville | Sparta |
|---|---|---|---|
| Metro area population (*persons*) | 403,892 | 9,269 | 1,770 |
| Study area population (*persons*) | 10,929 | 9,269 | 1,770 |
| Study area size (*km*$^2$) | 6.2 | 11.9 | 6.2 |
| Population density (*persons/mi*$^2$) | 1,731 | 778 | 285 |
| Development context | Urban | Suburban | Rural |
| Planning scale | Small-area plan | Comprehensive plan | Corridor plan |
| Geographic region | Piedmont | Coastal | Mountains |
| Proposed improvements | New sidewalks | New sidewalks | Streetscape improvements |
| Length of proposed improvements *(km)* | 30.9 | 82.7 | 0.6 |

**Table S2.** BRRC focus groups

| Meeting Date | Number of Participants | Stakeholder Affiliation |
|---|---|---|
| 2/28/2012 | 6 | BRRC residents |
| 3/1/2012 | 9 | BRRC HIA advisory council |
| 3/6/2012 | 7 | BRRC resident and property owners |
| 3/8/2012 | 12 | Employees and volunteers of the North Carolina Museum of Art |
| 3/20/2012 | 6 | Local officials, employees, local business owners, and students |

**Table S3.** Winterville and Sparta meeting participants

| | Participant | Role | Organization |
|---|---|---|---|
| Winterville | Alan Lilley | Planning Director | City of Winterville |
| | Jo Morgan | Health Education Director | Pitt County |
| | James Rhodes | Planning Director | |
| | Jennifer Smith | Manager | Vidant Health |
| | Daryl Vreeland | Transportation Planner | MPO |
| Sparta | Teresa Buckwalter | Principal | Consultant |
| | Eric Woolridge | Principal | |
| | Kevin Dowell | Planner and Codes Enforcement | Town of Sparta |
| | Bryan Edwards | Town Manager | |
| | Beth Fornadley | District Health Educator | Appalachian District Health Department |
| | Jennifer Greene | Director of Allied Health Services | |
| | Rachel Miller | CTG Health Eating/Active Living Lead | |
| | Jane Wyatt | Board Member | Chamber of Commerce |

**Table S3.** Summary of BRRC focus groups and Winterville and Sparta community meeting

| | **BRRC** (top twelve recommended changes from focus groups meetings) | **Winterville** | **Sparta** |
|---|---|---|---|
| Built environment and land use | ▪ Make the neighborhood more aesthetically pleasing<br>▪ Build more things to walk to<br>▪ Encourage mixed-use development<br>▪ Encourage greater land-use density | ▪ Non-walkable development scales<br>▪ Car-oriented development<br>▪ Segregated land uses<br>▪ Lack of services and employment within city<br>▪ School siting | ▪ Incomplete sidewalk network<br>▪ Heavy traffic along key routes<br>▪ Segregated land uses<br>▪ Rural school siting |
| Transportation infrastructure | ▪ Build sidewalks and crosswalks on major roads<br>▪ Build bike lanes and bike racks<br>▪ Build more walking trails<br>▪ Improve access to walking trails and open space<br>▪ Improve publicity of existing facilities (e.g., signage, maps, etc.) | ▪ Lack of sidewalks<br>▪ Poor sidewalk connections between developments<br>▪ Road widening projects undertaken without improvements to sidewalks/bike lanes<br>▪ Highway and rail that bisects town presents barriers to walking/biking<br>▪ Poor aesthetic quality of streets | ▪ Lack of sidewalks<br>▪ Width and quality of existing sidewalks (e.g., electric poles in the middle of sidewalks)<br>▪ Lack of zones to pass cyclists on rural roads<br>▪ Wide lanes throughout Sparta that encourage high travel speeds<br>▪ Downtown aesthetics not conducive to walking |
| Demographics and cultural factors | None | ▪ High rates of poverty<br>▪ High prevalence of risk factors (smoking, alcohol consumption, etc.) | ▪ High rates of poverty<br>▪ Older population<br>▪ Many residents do not have health insurance<br>▪ Cultural bias towards the car (rural setting)<br>▪ Poor nutrition/access to healthy foods<br>▪ Cultural norms that support tobacco use |
| Services | ▪ Improve the connectivity of public transportation<br>▪ Build more water fountains and restrooms for walkers and runners | ▪ Lack of public transit<br>▪ Poor access to facilities that offer affordable healthcare | ▪ Lack of public transit service<br>▪ Fragmentation of government services downtown: historically housed in a single building and residents would park once in downtown and walk to other destinations; services now offered in different buildings and residents drive to each |
| Social and/or economic conditions | ▪ Improve educational opportunities | ▪ Stigmatization of walking and biking for transportation<br>▪ Poor awareness the rules of the road by drivers, cyclists, and pedestrians in multi-modal situations | ▪ Stigmatization of walking for transportation<br>▪ Large percentage of the population on fixed incomes<br>▪ Large number of seasonal workers |
| Natural environment | None | ▪ Noise and air pollution due to North Carolina Highway 11 | ▪ Extreme elevation changes make cycling (walking not mentioned) difficult; thus, cycling is largely a recreational activity |

**Figure S4.** Case Study Population Distributions

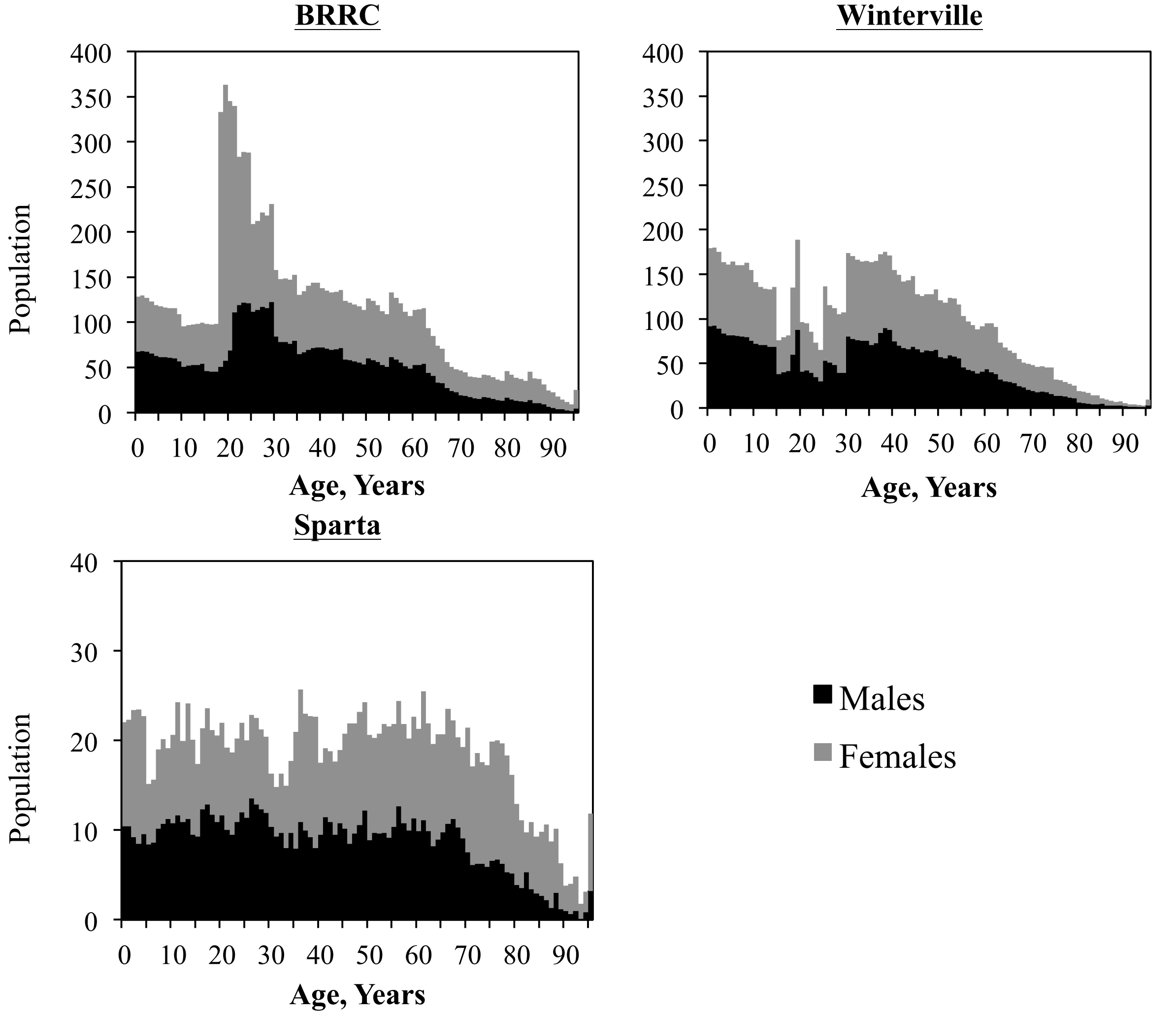


**S.2 Baseline Health Information**

Additional details are presented below regarding our procedure to estimate continuous disease prevalence and incidence functions for CHD, diabetes, hypertension, and stroke as a function of age in each case study location (Table S4). Detailed vital statistics (baseline death rate, birthrate, and gender ratio) are presented in Table S5.

**S.2.1 Disease Prevalence and Incidence Functions**

To develop continuous age- and sex-specific prevalence functions for CHD, diabetes, hypertension, and stroke, we use data from the 2009 North Carolina BRFSS survey. The survey asks whether or not a respondent has been diagnosed with these conditions and reports prevalence by age group. In each community, we fit a second-order function to these data assuming that the prevalence reported for each age group represented the actual prevalence of that disease at the population-weighted midpoint of the age group. Using these prevalence

estimates, we then derive the age-specific rate at which individuals would have had to develop a disease in order for the observed prevalence to occur. To do so, we define second-order age-specific prevalence functions, $p(x)$, and take the derivative:

$$p(x) = \alpha \cdot x^2 + \beta \cdot x + \gamma$$

$$\frac{dp}{dx} = 2 \cdot \alpha \cdot x + \beta$$

| | |
|---|---|
| $x$ = | age (years) |
| $\alpha$ = | derived parameter for second-order term |
| $\beta$ = | derived parameter for first-order term |
| $\gamma$ = | derived constant |

And define $c(x)$:

$$c(x) = \frac{\frac{dp}{dx}}{(1 - p(x))}$$

| | |
|---|---|
| $c(x)$ = | number of cases at age $x$ |

And define the incidence function, $i(x)$:

$$i(x) = c(x) + m(x) \cdot (1 - (p(x) \cdot R(x) - 1)^{-1})$$

| | |
|---|---|
| $i(x)$ = | Incidence rate at age $x$ |
| $m(x)$ = | All-cause mortality at age $x$ |
| $R(x)$ = | Relative risk of all-cause mortality associated with the disease for which incidence is being derived at age $x$ |

Estimated disease prevalence and incident functions are presented in Table S4.

**Table S4.** Baseline Disease Functions

| | Case Study Location | Prevalence as a function of age, *p(x)*<br>Incidence as a function of age, *i(x)* |
|---|---|---|
| **CHD** | BRRC | $p(x) = 9.7\times10^{-3} - 9.1\times10^{-4}x + 2.5\times10^{-5}x^2$<br>$i(x) = 0.37 - 5.0\times10^{-2}x + 2.4\times10^{-3}x^2 - 4.3\times10^{-5}x^3 + 2.8\times10^{-7}x^4$ |
| | Winterville | $p(x) = 6.1\times10^{-3} - 2.1\times10^{-4}x + 1.2\times10^{-5}x^2$<br>$i(x) = 0.38 - 4.5\times10^{-2}x + 2.0\times10^{-3}x^2 - 3.5\times10^{-5}x^3 + 2.3\times10^{-7}x^4$ |
| | Sparta | $p(x) = -2.3\times10^{-2} + 5.1\times10^{-4}x + 1.9\times10^{-5}x^2$<br>$i(x) = 0.50 - 4.8\times10^{-2}x + 2.2\times10^{-3}x^2 - 3.8\times10^{-5}x^3 + 2.5\times10^{-7}x^4$ |
| **Diabetes** | BRRC | $p(x) = -5.6\times10^{-2} + 2.1\times10^{-3}x + 1.1\times10^{-5}x^2$<br>$i(x) = 0.76 - 6.5\times10^{-2}x + 2.8\times10^{-3}x^2 - 5.1\times10^{-5}x^3 + 3.3\times10^{-7}x^4$ |
| | Winterville | $p(x) = -1.4\times10^{-2} - 3.9\times10^{-4}x + 4.4\times10^{-5}x^2$<br>$i(x) = 0.94 - 1.1\times10^{-1}x + 4.6\times10^{-3}x^2 - 8.0\times10^{-5}x^3 + 5.1\times10^{-7}x^4$ |
| | Sparta | $p(x) = -7.7\times10^{-2} + 3.4\times10^{-3}x + 1.3\times10^{-6}x^2$<br>$i(x) = 1.02 - 8.1\times10^{-2}x + 3.3\times10^{-3}x^2 - 5.5\times10^{-5}x^3 + 3.4\times10^{-7}x^4$ |
| **Hypertension** | BRRC | $p(x) = -7.6\times10^{-2} + 5.0\times10^{-3}x + 6.1\times10^{-5}x^2$<br>$i(x) = 2.3 - 2.1\times10^{-1}x + 9.6\times10^{-3}x^2 - 1.8\times10^{-4}x^3 + 1.2\times10^{-6}x^4$ |
| | Winterville | $p(x) = -2.1\times10^{-1} + 1.1\times10^{-2}x - 2.9\times10^{-6}x^2$<br>$i(x) = 2.7 - 2.0\times10^{-1}x + 8.9\times10^{-3}x^2 - 1.6\times10^{-4}x^3 + 1.0\times10^{-6}x^4$ |
| | Sparta | $p(x) = -1.6\times10^{-1} + 8.9\times10^{-3}x + 1.3\times10^{-5}x^2$<br>$i(x) = 1.8 - 1.1\times10^{-1}x + 5.1\times10^{-3}x^2 - 8.8\times10^{-5}x^3 + 5.9\times10^{-7}x^4$ |
| **Stroke** | BRRC | $p(x) = 2.9\times10^{-2} - 2.5\times10^{-3}x + 5.2\times10^{-5}x^2$<br>$i(x) = 1.3 - 1.5\times10^{-1}x + 6.3\times10^{-3}x^2 - 1.1\times10^{-4}x^3 + 6.6\times10^{-7}x^4$ |
| | Winterville | $p(x) = 3.1\times10^{-2} - 2.4\times10^{-3}x + 4.3\times10^{-5}x$<br>$i(x) = 2.5 - 2.7\times10^{-1}x + 1.0\times10^{-2}x^2 - 1.6\times10^{-4}x^3 + 9.0\times10^{-7}x^4$ |
| | Sparta | $p(x) = -1.3\times10^{-3} - 1.5\times10^{-4}x + 1.5\times10^{-5}x$<br>$i(x) = 0.52 - 5.9\times10^{-2}x + 2.6\times10^{-3}x^2 - 4.6\times10^{-5}x^3 + 3.0\times10^{-7}x^4$ |

**Table S5.** Baseline Vital Statistics

| | | BRRC | | Winterville | | Sparta | |
|---|---|---|---|---|---|---|---|
| | Age Group | Male | Female | Male | Female | Male | Female |
| Death Rate (per 100,000) | 0-5 | 160.01 | 172.81 | 226.60 | 243.86 | 367.65 | 75.71 |
| | 5-10 | 6.63 | 13.79 | 57.45 | 20.52 | 188.39 | 94.80 |
| | 10-15 | 16.65 | 7.00 | 20.69 | 0 | 331.13 | 118.69 |
| | 15-20 | 49.94 | 19.61 | 61.51 | 13.58 | 286.16 | 148.61 |
| | 20-25 | 93.44 | 27.91 | 152.66 | 30.10 | 352.67 | 186.08 |
| | 25-35 | 80.80 | 31.83 | 186.20 | 77.9 | 146.41 | 378.07 |
| | 35-45 | 115.57 | 89.44 | 187.38 | 117.32 | 787.40 | 408.71 |
| | 45-55 | 245.93 | 182.33 | 744.58 | 352.75 | 626.57 | 641.85 |
| | 55-65 | 727.96 | 530.22 | 1,088.58 | 643.99 | 985.22 | 853.66 |
| | 65-75 | 2,079.77 | 1,508.45 | 3,381.39 | 2,321.51 | 2,503.91 | 845.07 |
| | 75-85 | 5,955.81 | 4,021.64 | 6,068.60 | 4,555.74 | 5,507.25 | 1,486.20 |
| | 85+ | 14,704.68 | 14,568.07 | 14,951.77 | 12,741.31 | 11,764.71 | 9,691.63 |
| Birth Rate | | 0.0146 | | 0.0145 | | 0.00977 | |
| Gender Ratio (M:F) | | 1.05 | | 1.04 | | 1.25 | |

**S.3 Baseline Transportation Behavior**

In Winterville and Sparta, we use data from the 2009 BRFSS survey. In 2009, North Carolina included an additional question regarding walking for transportation. Specifically, the survey asked “In the past week, how much time did you walk or bicycle for transportation, such as to and from work or shopping, or walk to the bus stop?” Respondents replied in one of five categories: No time, Less than 30 minutes, 30 minutes to 1 hour, 1 to 2 hours, or 2 hours or more.[34] In Winterville, we use county-level data (Pitt County) whereas in Sparta we use data aggregated across the Northwest Area Health Education Center (HEC), a ten-country area (Alleghany, Ashe, Davie, Davidson, Forsyth, Stokes, Surry, Watauga, Wilkes, and Yadkin counties). In BRRC, we use data from a survey conducted in 2012 by MacDonald Gibson et al. The survey used the International Physical Activity questionnaire, a previously validated survey instrument.[37] The survey asked two questions from which estimates of weekly walking for transportation were derived: “During the last 7 days, on how many days did you walk for at least 10 minutes at a time to go from place to place?” immediately followed by “How much time did you usually spend on one of those days walking from place to place?” These estimates were then used to develop a distribution of walking for transportation time by placing each in one of 20 transportation physical activity time bins to: one for no walking, a series of twenty-minute bins up to 360 minutes per week (i.e., 0–20 minutes, 20–40 minutes, etc.), and a top bin for greater than 360 minutes per week.[36] Survey characteristics are summarized in Table S6.

**Table S6.** Baseline Transportation Physical Activity Survey Characteristics

| Case Study Location | Survey and question wording | Sample size | Responses Category | n | Percent |
|---|---|---|---|---|---|
| BRRC | Survey Based on International Physical Activity Questionnaire<br><br>*Question wording: "During the last 7 days, on how many days did you walk for at least 10 minutes at a time to go from place to place?*<br><br>*How much time did you usually spend on one of those days walking from place to place?"* | 386 | 0 | 157 | 40.7% |
| | | | 1–20 | 28 | 7.3% |
| | | | 20–40 | 30 | 7.8% |
| | | | 40–60 | 32 | 8.3% |
| | | | 60–80 | 17 | 4.4% |
| | | | 80–100 | 21 | 5.4% |
| | | | 100–120 | 18 | 4.7% |
| | | | 120–140 | 8 | 2.1% |
| | | | 140–160 | 7 | 1.8% |
| | | | 160–180 | 6 | 1.6% |
| | | | 180–200 | 1 | 0.3% |
| | | | 200–220 | 13 | 3.4% |
| | | | 220–240 | 3 | 0.8% |
| | | | 240–260 | 2 | 0.5% |
| | | | 260–280 | 7 | 1.8% |
| | | | 280–300 | 4 | 1.0% |
| | | | 300–320 | 4 | 1.0% |
| | | | 320–340 | 0 | 0.0% |
| | | | 340–360 | 4 | 1.0% |
| | | | 360+ | 24 | 6.2% |
| Winterville (Pitt County) | 2009 NC BRFSS<br>*Question wording: "In the past week, how much time did you walk or bicycle for transportation, such as to and from work or shopping, or walk to the bus stop?"* | 323 | 0 | 276 | 84.3% |
| | | | 1–30 | 14 | 3.4% |
| | | | 30–60 | 11 | 2.5% |
| | | | 60–120 | 9 | 2.9% |
| | | | 120+ | 13 | 6.9% |
| Sparta (Northwest Area HEC) | 2009 NC BRFSS<br>*Question wording: "In the past week, how much time did you walk or bicycle for transportation, such as to and from work or shopping, or walk to the bus stop?"* | 2,661 | 0 | 2,322 | 85.3% |
| | | | 1–30 | 82 | 3.7% |
| | | | 30–60 | 70 | 3.2% |
| | | | 60–120 | 70 | 2.7% |
| | | | 120+ | 117 | 5.0% |

**S.4 Economic Valuations**

To account for uncertainty inherent in selecting an appropriate discount rate, we consider three discount rates: 7%, 5%, and 3.5%. Benefit-cost ratios for the central estimate of health outcomes for each case study location at each of these three discount rates are plotted in Figure S2.

**Table S7.** Economic valuation assumptions

| Health Outcome | Source of Monetary Benefits | Monetary Value (2012 USD) |
|---|---|---|
| Avoided premature mortality | Value of a statistical life (VSL) | $9,100,000 |
| | *Yearly treatment costs* | *$8,154* |
| CHD | *Yearly productivity losses* | *$4,981* |
| | Total yearly costs avoided: | $13.135 |
| | *Yearly treatment costs* | *$11,508* |
| Diabetes | *Yearly productivity losses* | *$2,763* |
| | Total yearly costs avoided: | $14.271 |
| | *Yearly treatment costs* | *$11,321* |
| Hypertension | *Yearly productivity losses* | *$1,265* |
| | Total yearly costs avoided: | $12,685 |
| | *Yearly treatment costs* | *$13,551* |
| Stroke | *Yearly productivity losses* | *$9,001* |
| | Total yearly costs avoided: | $22,552 |

**Figure S5.** Economic valuations over time

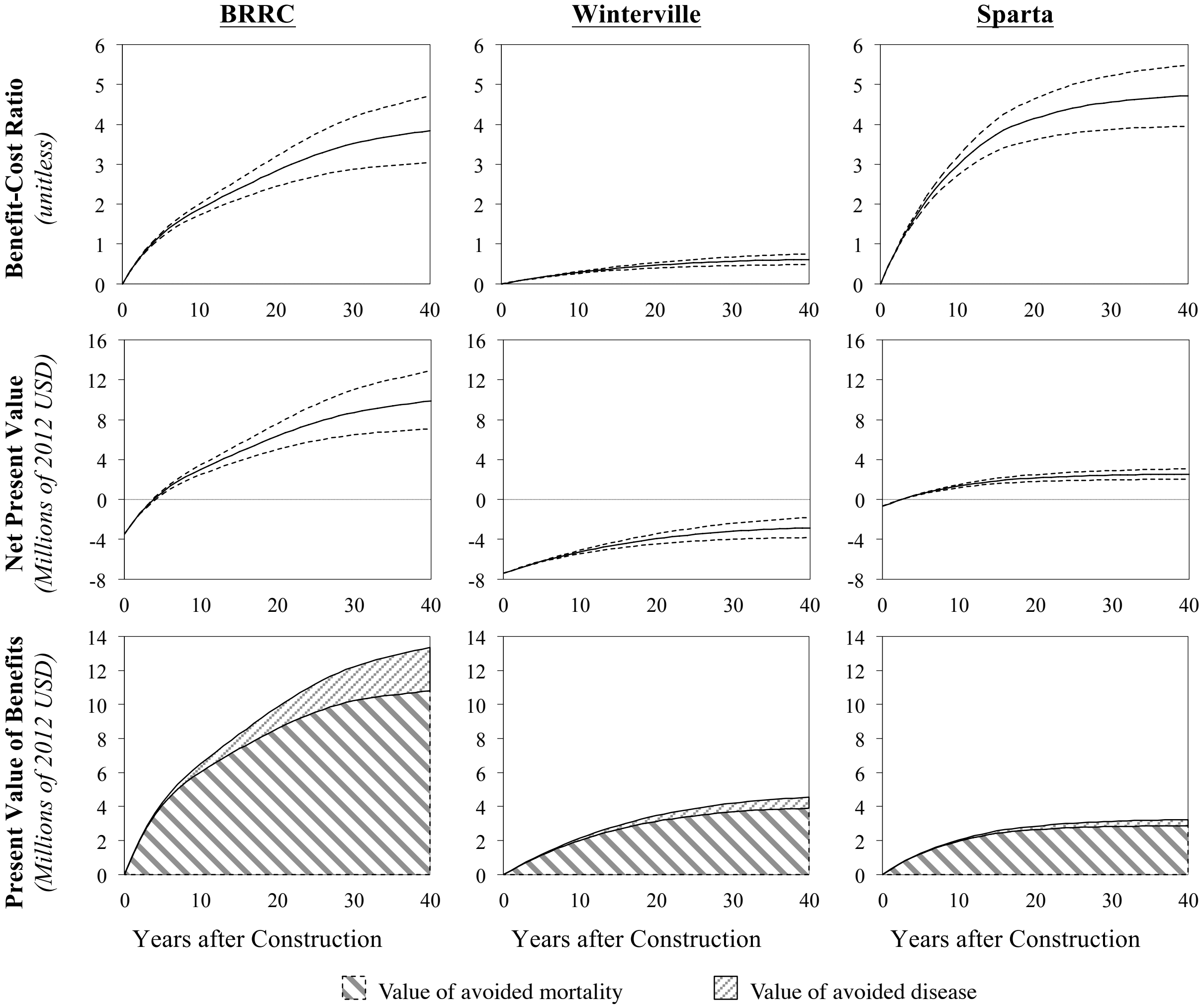

Supplement: Supplementary file 1 — The Supplementary Material includes four sections. Section 1 provides additional details on each community, descriptions of proposed pedestrian infrastructure improvement projects, and summaries of community meetings. Section 2 presents the procedure used to estimate disease incidence functions and provides additional detail on baseline health data. Section 3 summarizes the surveys used to characterize preconstruction walking for transportation. Finally, Section 4 provides the economic values of health outcomes used in analysis and plots of economic valuations over time for each community. [file 812325.f1.pdf]
